# Chronic allograft rejection: A significant hurdle to transplant success

**DOI:** 10.4103/2321-3868.121646

**Published:** 2014-01-26

**Authors:** Malgorzata Kloc, Rafik M. Ghobrial

**Affiliations:** 1 Department of Surgery, Houston Methodist Hospital, Houston, USA; 2 Houston Methodist Hospital Research Institute, Houston, TX USA; 3 Department of Surgery, The Methodist Hospital, 6550 Fannin St., Houston, TX 77030 USA

**Keywords:** Chronic rejection, macrophage, neointima, consists of smooth muscle cells, vasculopathy

## Abstract

The state-of-the-art immunosuppression drugs do not ensure indefinite transplant survival, and most transplants are continuously lost to chronic rejection even years posttransplantation. This form of rejection is responsible for long-term failure of transplanted organs. The mechanisms involved in development of chronic rejection are not well-understood. One of the main features of chronic rejection is progressive luminal narrowing of graft vessels, which results in compromised blood flow, ischemia, cell death, and finally graft failure. All the existing immunosuppressive regimens are targeting acute rejection, and at present there is no available therapy for prevention of chronic rejection. Chronic rejection involves two major, but interrelated responses: The first is the host immune response against the transplant mediated primarily by alloreactive T and B cells, and the second is injury and repair of the graft (vasculopathy of graft vessels). Here we focus on recent advances in understanding the cellular and molecular aspects of chronic transplant vasculopathy and function of macrophages, topics pivotal for development of novel antichronic rejection therapies.

## Introduction

There are three major types of allograft rejection: Hyperacute, acute, and chronic rejection.[1] Hyperacute rejection occurs within minutes and hours after transplantation and is caused by the presence of preexisting antidonor antibodies in the recipient blood. Recognition of donor antibody activates the complement system, induces influx of neutrophils, and promotes coagulation. The resulting inflammation and ischemia induce irreversible damage of the graft. Fortunately, existing screenings for antidonor antibodies eliminate most of hyperacute rejection cases. Acute rejection occurs within the first weeks to several months after transplantation and usually affects every transplanted organ to some degree. Acute rejection is caused by the mismatch in highly polymorphic human leukocyte antigens (HLA) and is mediated primarily by T cells. They produce cytokines upon activation, which recruit inflammatory cells eventually leading to necrosis of graft tissue. Presently, the acute rejection can be successfully contained by immunosuppressive therapies.

**Table 1:** 

Access this article online	
**Quick Response Code:**	**Website:** www.burnstrauma.com
	**DOI:** 10.4103/2321-3868.121646

Chronic rejection develops within months to years after transplantation and is the major cause of long-term graft loss. The main feature of chronic rejection is accelerated arteriosclerosis or progressive luminal narrowing of graft vessels (vasculopathy or graft vascular disease (GVD)) often accompanied by graft tissue (parenchymal) fibrosis. These, in turn, result in ischemia, cell death, and graft failure. All the existing immunosuppressive regimens are highly focused on prevention of acute rejection, and at present there are no available antichronic rejection therapies. Thus, understanding the cellular and molecular events contributing to chronic rejection of transplanted organs is pivotal for development of novel antichronic rejection therapies.

## Transplant vasculopathy

One of the most distinctive features of chronic rejection of heart, kidney, liver, and lung allografts is the progressive occlusion or intimal hyperplasia of the blood vessels, which compromises blood flow and results in ischemia and eventual failure of the graft.[2]

**Figure 1:**
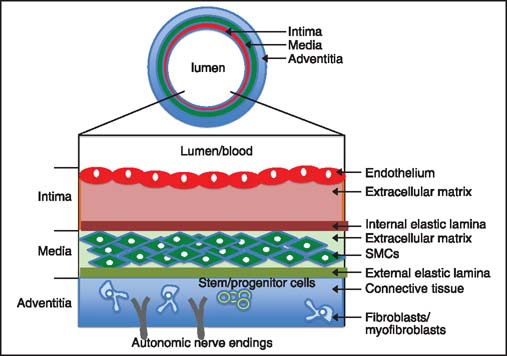
Blood vessel composition. Large blood (arteries and veins) vessels are composed of three layers (shown here out of proportion). Tunica intima (the thinnest layer, facing the blood) contains a single layer of endothelial cells positioned on subendothelial extracellular matrix and circularly arranged elastic bands called the internal elastic lamina. Tunica media (which is the thickest layer in arteries) is composed of extracellular matrix, smooth muscle cells (SMCs) and thick elastic band called external elastic lamina. Tunica adventitia (which is the thickest layer in veins) is made of connective tissue with interspersed fibroblasts and stem/progenitor cells. It also contains nerve endings and nutrient capillaries (vasa vasorum) in the larger blood vessels.

Normal blood vessel is built of several distinct layers [Figure 1].[1] The most inner (facing the lumen/blood) and also the thinnest layer called the tunica intima (or intima) consists of a single layer of endothelial cells positioned on a layer extracellular matrix composed of collagen and proteoglycans and a sheet of elastic fibers called the internal lamina. The second (middle layer) called the tunica media (or media) consists of smooth muscle cells (SMCs) embedded in elastin-rich extracellular matrix and positioned on external elastic lumina [Figure 1]. The outer layer, the tunica adventitia (also called tunica externa or adventitia) is built of connective tissue with interspersed fibroblasts, quiescent resident inflammatory cells, myofibroblasts, recently identified large population of resident stem/progenitor cells[34] and autonomic nerve endings [Figure 1].[1,5–7] All these constituents of blood vessels undergo profound remodeling during chronic rejection.

## Vascular remodeling in chronic rejection

**Figure 2:**
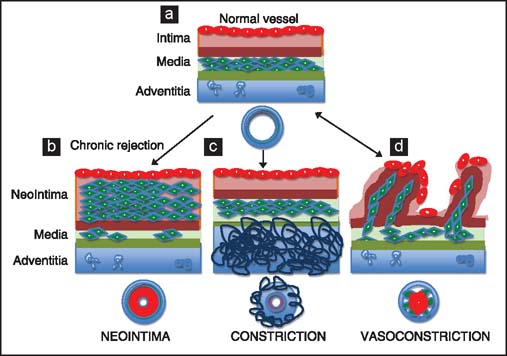
Vascular remodeling: (a) Diagram of normal blood vessel. (b) Thickening of the intima, through the recruitment and proliferation of smooth muscle cells, is dominant form of vascular remodeling in chronic rejection. (c) Constrictive remodeling caused by a healing response and formation of scar tissue in the adventitia results in squeezing the media and intima inward. (d) Vasoconstriction is caused by reversible over contraction of smooth muscle cells in the tunica media.

Although the narrowing of vessel lumen can be caused by one of the three major types of vascular remodeling: Thickening of the intima (through the recruitment of extraneous smooth muscle cells and some leukocytes), constrictive remodeling (when a healing response in the adventitia results in the formation of collagen-rich scar tissue, which squeezes the media and intima inward),[8] and vasoconstriction (a hypercontraction of smooth muscle cells in the tunica media) [Figure 2]; the intimal thickening (formation of neointima) seems to play a dominant role in vascular remodeling during chronic rejection.[1,2,5–7]

**Figure 3:**
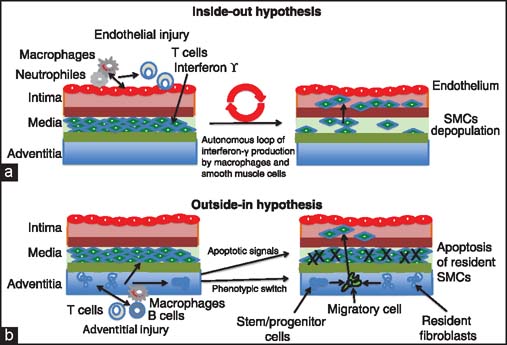
Hypotheses of vascular injury. (a) In the “inside-out” hypothesis the first target of injury is vascular endothelium, which produces inflammatory and adhesion molecules leading to the accumulation of leukocytes (macrophages, neutrophils and interferon-γ producing T cells). Endothelial cells and activated macrophages produce growth factors, which together with interferon-γ produced by T cells (and subsequently in autonomic loop by macrophages and SMCs), stimulate the SMCs residing in the media to acquire proliferative phenotype and migrate to the intima. This leads to progressive loss of SMCs from the media and thickening of the intima (formation of the neointima). (b) In the “outside-in” hypothesis the inflammatory response is initiated at the adventitia and progresses inward. Signaling from the inflamed adventitia leads to apoptosis of resident SMCs of media. In addition, it results in the production of cytokines and growth factors, which induce phenotypic switch of adventitia progenitor/stem cells and/ or resident fibroblasts into migratory cells; which migrate to the intima, proliferate, and differentiate into SMCs.

**Figure 4:**
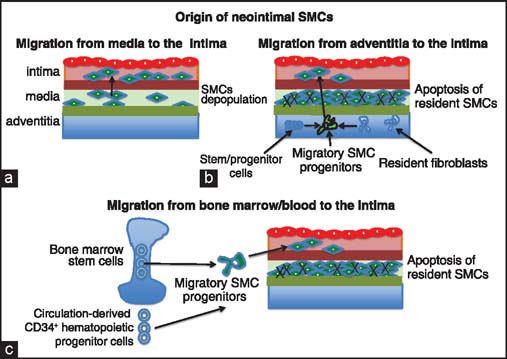
Hypothetical origin of neointimal SMCs. (a) SMCs of the media (after switching into migratory phenotype) transmigrate into newly forming neointima. (b) SMCs present in the media undergo apoptosis. Stem/progenitor cells and resident fibroblasts present in adventitia acquire migratory phenotype and transmigrate into intima where they proliferate and differentiate into SMCs. (c) SMCs present in the media undergo apoptosis. Bone marrow progenitor cells (and possibly also circulation-derived CD34^+^ hematopoietic progenitor cells) acquire migratory phenotype, migrate into the intima, proliferate, and differentiate into SMCs.

There are two major hypotheses concerning the initiation of vascular remodeling and inflammatory response. Traditional concept called the “inside-out” response assumes that the inflammatory response to various types of injuries (including immune injury during chronic rejection) is initiated at the endothelial layer of the intima, that is, at the luminal (inside) surface of the vessel [Figure 3]. According to this “inside-out” hypothesis the first target of chronic alloresponse and inflammatory response is the vascular endothelium, which produces cytokines, chemokines, and adhesion molecules leading to the accumulation of leukocytes (predominantly macrophages). In addition, oxidized lipids in the circulation accumulate in the macrophages, which are present on the surface of the intima leading to endothelial cells injury and initiation an inflammatory process. Subsequently, endothelial cells and activated macrophages produce growth factors, which together with interferon-γ produced by T cells (and subsequently in autonomic loop by macrophages and SMCs), stimulate SMCs of the media to change the phenotype [Figure 3].[3,4,9–13] After further stimulation, the SMCs residing in the media acquire proliferative phenotype and transmigrate into the subendothelial layer of intima leading to progressive decellularization of the media, thickening of the intima (formation of the neointima), and progressive occlusion of the vessel [Figures 3 and 4].[14,15] The fact that there is progressive loss (especially dramatic in chronic rejection) of the SMCs from the media and that neointimal cells express α-actin (which is a specific marker of smooth muscle cells) has been considered as a proof of principle that neointimal cells may actually derive from the SMCs of the donor vessel media.[9,11] Recently, there is mounting evidence supporting a new “outside-in” hypothesis in which the inflammatory response is initiated at the outer layer of the vessel, that is, at the adventitia and propagates inwards toward the intima [Figure 3]. According to this hypothesis the adventitial inflammation results in the production of cytokines and growth factors, which stimulate phenotypic switch of resident progenitor or stem cells and/or resident fibroblasts into migratory cells, which after migration into intima can differentiate into α-actin-producing SMCs [Figure 3].[3,4] Studies using the acute vascular injury models indicate that indeed the adventitia contains a resident population of vascular progenitor cells that are able to differentiate into SMCs and then migrate from the adventitia to the forming neointima [Figures 3 and 4]. [5,16]

## Origin of SMCs in intimal thickening

Although it is well-established that the thickening of the intima is the results of migration of extraneous cells into the intima [Figure 3]. In the past few decades, there has been much controversy about the source and identity of these cells. [3,5,11,17,18] The generally accepted hypothesis, mostly based on the response-to-injury model of host native atherosclerosis setting, assumes that both in “inside-in” and “outside-in” scenario the SMCs of neointima originate from the vessel wall (either from the media or from adventitia) of the donor vessel. However, the assumption that SMCs originate exclusively from the donor has been recently challenged [Figure 4].[5,11,18] Using mouse vascular mechanical injury as a model of neointima formation in native atherosclerosis, Han *et al.*,[19] showed that neointimal cells can derive (at least partially) from the recipient bone marrow cells expressing smooth muscle specific α-actin. Although these data support the hypothesis of recipient origin of neointimal cells in native atherosclerosis, they do not prove that the same process occurs during chronic transplant rejection. To address this issue, Skaro *et al.*,[11] attempted to address the mechanism responsible for the loss of SMCs from the media during chronic rejection. The question the authors wanted to resolve is whether the loss of SMCs within the media is due to *in situ* destruction or their transmigration from the media to the neointima. This study showed that the loss of cells from the media of allograft vessels is the result of cytolytic cell-induced apoptosis, but not depopulation resulting from SMC transmigration.[11] In addition, studies on nonimmunosupressed and cyclosporin (CsA) suppressed rat aortic allografts showed that the cells present in allograft neointima originate from the recipient.[11,20,21] These results created new questions: What is the source and identity of recipient cells that accumulate in the graft neointima and what is their route of entry into the allograft intima? Although some of the studies in rodent and human allografts show that neointimal SMCs originating from the circulating mesenchymal progenitor cells probably derived from the recipient bone marrow [Figure 4],[22–24] other studies indicate that the bone marrow is not a major source of cells populating graft neointima[19,20,23] and that circulation-derived CD34^+^ hematopoietic progenitor cells give rise to smooth muscle progenitor cells that may eventually migrate to the graft vessels.[18,25] Interestingly, several recent studies on the origin of the SMCs conclude that the bone marrow-derived cells do not participate in neointima formation,[5,26,27] and point to the arterial adventitia as the major source of neointimal SMCs.[16,28] In view of this persisting controversy [Figure 4], it still remains to be defined how various cell types, both donor and host origin, contribute to the formation of neointima during chronic allograft rejection. This is a significant issue that warrants further investigation.

## Migration and proliferation of SMCs

**Figure 5:**
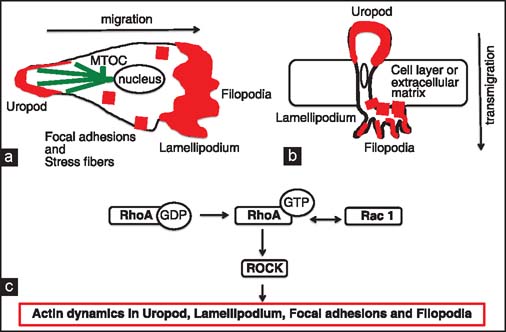
Actin- and RhoA/Rac1 pathway-dependent functions in cell migration and transmigration. (a) Migratory and (b) transmigratory phenotypes of cells such as smooth muscle cells (SMCs), fibroblasts, or macrophages; depend on the polarization of microtubules emanating from centriole-containing MTOC (or centrosome) and the formation of actin-rich (shown in red) leading (lamellipodium) and trailing (uropod) edge. Lamellipodium is able to form, spiky, and actin-rich projections called filopodia that play a role in sensing of the environment and adhesion (together with actin rich focal adhesions and/or stress fibers) to the substratum. Mother and daughter centriole of MTOC and microtubules are shown in green. MTOC-derived microtubules deliver, via function of molecular motors, various molecules (such as actin-binding proteins) and vesicles to proper destinations within the cell. (c) Actin dynamics (such as polymerization, depolymerization, and binding to its partners) in uropod, lamellipodium, filopodia, and focal adhesion/stress fibers are regulated by interplay between small GTPase RhoA and Rac1 pathways.[59]

**Figure 6:**
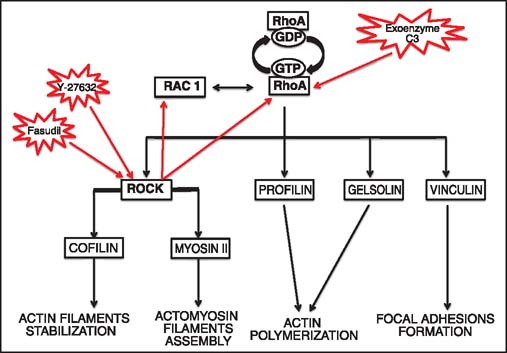
RhoA/Rac1 pathways regulate actin dynamics. RhoA and its downstream effector ROCK, and their reciprocal interaction with Rac1, regulate (through actin-binding partners: Cofilin, myosin II, profilin, and vinculin) the pleiotropic actin-dependent processes pivotal for cell movement, phagocytosis, and cell differentiation. RhoA pathway inhibitors such as Y-27632, exoenzyme C3, and fasudil inhibit SMCs migration and may prevent chronic rejection-related vasculopathy.

Although very little is known about the route and mechanisms, operating in the migration of SMCs into the intima during chronic rejection per se, a plethora of information on the SMC movement in various model systems may apply to the SMC movement during vessel wall remodeling in chronic rejection. It is well-established that the cell migration starts when cell surface receptors are stimulated, which, in turn, initiates a cascade of cytoskeleton remodeling events such as actin polymerization, repositioning of the microtubule organizing center (MTOC), changing the adhesiveness to the substratum, and eventually the emergence of the actin-rich leading and trailing edge (uropod) of the motile cell [Figure 5].[18,29,30] Numerous studies indicate that, in majority of cell types, cell adhesion and motility (through the actin cytoskeleton remodeling) are under the control of pleiotropic small GTPase RhoA/ROCK and Rac1 pathways [Figures 5 and 6].[31] Accordingly, it has been also shown that RhoA activates ROCK1 and ROCK2 in SMCs[32] and that the ROCK1 inhibitor Y-27632, RhoA/RhoB/RhoC inhibitor C3 exoenzyme, and ROCK inhibitor fasudil block SMCs migration.[33,35] In addition, studies on ROCK1+/-mice indicate that ROCK1 is required for the development of cardiac fibrosis and is also involved in myocyte differentiation. The p190-B Rho GAP-deficient mice with chronically activated RhoA/ROCK pathway have predilection toward myocyte differentiation and the treatment of these mice with the Y-27632 inhibitor down regulates myocyte pathway[32,36] Studies from our laboratory showed that Y-27632 inhibitor abrogates chronic rejection of cardiac allografts in a rat model and inhibits intimal thickening of the allograft vessels.[37] These findings suggest that this inhibitor prevents or disrupts migration of SMCs into the intima. Interestingly, statins, which are used for treatment of hyperlipidemias by affecting synthesis of small GTPase proteins, also inhibit SMC proliferation and migration.[38,39]

It is well-established that after migration to the intima SMCs undergo extensive proliferation. Although the signaling pathways responsible for SMCs proliferation during alloimmune response are not fully understood, it seems that platelet-derived growth factor (PDGF) is one of the regulators involved.[40,41] Recent studies of chronic allograft vasculopathy demonstrated that the enzyme transglutaminase 2 (TG2)-mediated activation of β-catenin signaling plays a major role in proliferation of neointimal SMCs in murine cardiac allograft system.[42]

Over the years, several therapeutically important drugs have been identified that prevent vessel wall thickening by either inhibiting SMC migration or inhibiting cell cycle-progression, and thus decreasing SMC proliferation or both. For example, sabiporide, a new Na/H exchanger inhibitor,[43] rapamycin,[44] taxol,[45] and antidiabetic and anti-inflammatory drug troglitazone,[46] all inhibit cell cycle progression and SMC migration. However, although these drugs are often beneficial in preventing vessel remodeling during atherosclerotic disease in humans and some, like Y-27632 inhibitor, block chronic rejection in rodents.[37,47,48] So far none have been proven effective in inhibition of chronic rejection in humans. This underscores the fact that chronic rejection is far more complex than simple SMC migration and proliferation in the vessel intima and that chronic rejection prevention or treatment will probably require complex and multitargeted approaches, including the manipulation of immune cells and immunological response. One of the most recent promising cell types, whose modulation may improve allograft functions, is the macrophages.

## Macrophages and graft injury

**Figure 7:**
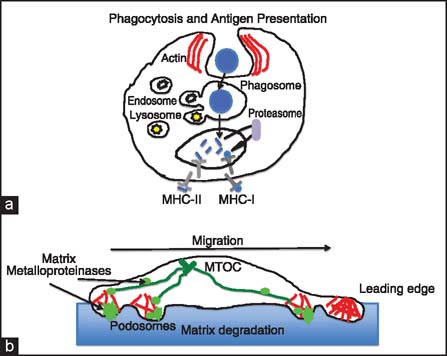
Macrophage actin-pendent functions. (a) Macrophage phagocytosis and down-the-road antigen presentation depend on the actin cytoskeleton. Engulfment (phagocytosis) of foreign material, such as bacteria, viruses, dead cells, or cell debris is facilitated by the actin-myosin contractile-dependent phagosome formation at the cell surface. Once inside the cytoplasm, phagosome (containing ingested material) is then fused with the endosomes and lysosomes, leading to ingested material degradation and/or further processing into peptides through the proteasomal pathway. The resulting antigenic peptides are presented on the cell surface by major histocompatibility complex (MHC) class I and/or MHC class II molecules. (b) Macrophage migration not only depends on the formation and function of actin rich leading and trailing edge, but also on their ability to digest and degrade extracellular matrix. Extracelluar matrix degradation depends on the function of actin- and metalloproteinase-rich organelles called the podosomes. Podosomes play a role in cellular motility within tissue by coordination of degradation of the extracellular matrix with cellular movement. In macrophages the podosomes are either dispersed on the cell surface or they are clustered into higher order complex called the podosomal rosette. Podosomal metalloproteinases (shown in light green), which are used for matrix degradation, are delivered to the podosomes via microtubules emanating from microtubule organizing center (MTOC) (shown in dark green) and molecular motors.[60]

Macrophages are one of the most plastic and mysterious cell types of immune response and allograft injury. Macrophages are professional phagocytes and antigen-presenting cells [Figure 7], which differentiate from circulating peripheral blood monocytes. In response to different cytokine signals they differentiate into classically activated M1-inflammatory macrophages (CAM) or alternatively activated anti-inflammatory M2 macrophages (AAM).[49,50] M1 macrophages are highly effective in phagocytosis, they produce high level of inducible nitric oxide synthase 2 (iNOS) and potent proinflammatory cytokines such as interleukin (IL)-1, IL-6, and IL-23; thus, may participate in allograft damage. M2 macrophages produce anti-inflammatory cytokines (IL-10), and growth factors (PDGF, transforming growth factor (TGF)-β, endothelial growth factor (EGF), vascular endothelial growth factor (VEGF), and chitinase and chitinase-like proteins involved in immunosuppression, extracellular matrix turnover, and tissue repair). M2 macrophages are also involved in angiogenesis and tissue remodeling, and therefore, they may promote allograft damage repair. Numerous studies showed that macrophages infiltrate allografts and allograft vessel intima during both acute and chronic rejection. In rodent models, depletion of macrophages inhibits transplant vasculopathy and improves allograft functions.[49,51,52] Recently, a new type of macrophage called regulatory macrophage or Mreg has been identified, which seem to play an antiinflammatory function. Recent pilot study of adoptive transfer of donor-derived Mregs into human recipients of kidney allografts demonstrated a dramatic lowering of the immunosuppression drugs in transplant patients and improved allograft outcome.[53,54] Whether this improved graft function is the result of reduced chronic rejection remains unknown.

Macrophages present within the transplanted organs derive from two main sources: 1. Donor-derived macrophages, which are present in the organ during transplantation, and 2. recipient-derived macrophages; which enter the graft after transplantation. Within first few weeks posttransplantation donor-derived macrophages proliferate, and subsequently, in non-rejecting graft, their number gradually declines. Studies on kidney transplant models showed that during acute rejection there are massive accumulation of recipient-derived macrophages and their proliferation within the allograft and thus amplify rejection response.[52] Although it remains unknown how exactly macrophages promote development of chronic rejection it seems that may promote cell death (through the production of nitric oxide), fibrosis (through the release of metalloproteases and (TGF)-β, smooth muscle proliferation (via release of platelet derived growth factor PDGF), and cytokine-mediated inflammation (via IL-1 and tumor necrosis factor alfa (TNF)-α.[52] Recent studies of the role of macrophages in chronic rejection in rat cardiac allograft model using noninvasive magnetic resonance imaging (MRI) tracking of macrophages labeled with micron-sized paramagnetic iron oxide particles showed that the number of recipient-derived macrophages increases during development of chronic rejection, and that persistence of macrophage accumulation after postacute rejection stage may be an indicator of developing chronic rejection (CR).[55]

**Figure 8:**
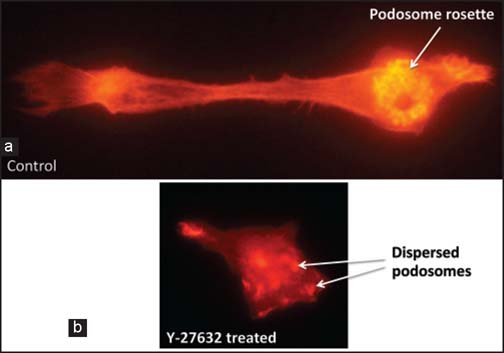
RhoA pathway inhibitor Y-27632 abrogates macrophage polarization and podosome formation. The unpublished data from our laboratory show that Y-27632 inhibitor prevents actin-dependent polarization of macrophage morphology and formation of actin-containing podosome rosette. (a) Mouse control macrophage shows highly elongated and polarized phenotype and the podosomes clustered into the rosette. (b) Macrophage incubated in the presence of Y-27632 inhibitor does not polarize and shows low number of dispersed podosomes. Actin was stained red with rhodamine-conjugated phalloidin. Yellow tint of podosome rosette in the control macrophage image results from the overexposure of highly concentrated actin in the podosomes.

Macrophage functions such as cell polarity, motility, interactions with extracellular matrix, phagocytosis, and antigen presentation; are all actin cytoskeleton-dependent, and as such they are regulated by small GTPase RhoA/ROCK and Rac1 pathways [Figures 3, 6 and 7].[56,58] This, in turn, suggests that inhibitors of these pathways could be potentially used to modulate macrophage functions in chronic rejection. Indeed, recent (unpublished) studies from our laboratory indicate that RhoA pathway inhibitor Y-27632, which abrogates chronic rejection of heart allografts in rodent models, dramatically modifies macrophage morphology and polarization [Figure 8]. Thus, it seems that together with the SMCs, different aspects of macrophage-related alloimmune response may be promising targets for novel antichronic rejection therapies.

## Summary

Chronic allograft rejection is a major hurdle to transplant success in the clinic and overcoming this hurdle is critical to advance transplant medicine. One of the most striking features of chronic rejection is the concentric neointima formation affecting virtually all blood vessels in the transplanted organs. The mechanisms behind this distinct pathology remain incompletely defined and multiple mechanisms, pathways, and cell types have been implicated in this process. Further understanding of the fundamental mechanisms related to cell motility, migration, and polarization as well as tissue repair and remodeling may lead to the development of new therapies to treat or prevent chronic allograft rejection.

## References

[1] Mitchell RN (2009). Graft vascular disease: Immune response meets the vessel wall. Annu Rev Pathol Mech Dis.

[2] Julius BK, Attenhofer Jost CH, Sutsch G, Brunner HP, Kuenzli A, Vogt PR (2000). Incidence, progression and functional significance of cardiac allograft vasculopathy after heart transplantation. Transplantation.

[3] Hu Y, Xu Q (2011). Adventitial Biology: Differentiation and Function Arterioscler. Thromb Vasc Biol.

[4] Maiellaro K, Taylor WR (2007). The role of the adventitia in vascular inflammation. Cardiovasc Res.

[5] Hoglund VJ, Dong XR, Majesky MW (2010). Neointima formation: A local affair. Arterioscler Thromb Vasc Biol.

[6] Lusis AJ (2000). Atherosclerosis. Nature.

[7] Libby P, Pober JS (2001). Chronic rejection. Immunity.

[8] Schwarzacher SP, Uren NG, Ward MR, Schwarzkopf A, Giannetti N, Hung S (2000). Determinants of coronary remodeling in transplant coronary disease: A simultaneous intravascular ultrasound and Doppler flow study. Circulation.

[9] Ross R (1993). The pathogenesis of atherosclerosis: A perspective for the 1990’s. Nature.

[10] Libby P (2000). Changing concepts in atherogenesis. J Int Med.

[11] Skaro AI, Liwski RS, Johnson P, Legare JF, Lee TD, Hirsch GM (2002). Donor versus Recipient: Neointimal Cell Origin in Allograft Vascular Disease Graft.

[12] Li AC, Glass CK (2002). The macrophage foam cell as a target for therapeutic intervention. Nature Medicine.

[13] Palinski W, Ord VA, Plump AS, Breslow JL, Steinberg D, Witztum JL (1994). ApoE-deficient mice are a model of lipoprotein oxidation in atherogenesis. Demonstration of oxidation-specific epitopes in lesions and high titers of autoantibodies to malondialdehydelysine in serum. Arterioscler Thromb.

[14] Akyurek LM, Paul LC, Funa K, Larsson E, Fellstrom BC (1996). Smooth muscle cell migration into intima and adventitia during development of transplant vasculopathy. Transplantation.

[15] Murry CE, Gipaya CT, Bartosek T, Benditt EP, Schwartz SM (1997). Monoclonality of smooth muscle cells in human atherosclerosis. Am J Pathol.

[16] Daniel JM, Bielenberg W, Stieger P, Weinert S, Tillmanns H, Sedding DG (2010). Time course analysis on the differentiation of bone marrow derived progenitor cells into smooth muscle cells during neointima formation. Arterioscler Thromb Vasc Biol.

[17] Kennedy LJ, Weissman IL (1971). Dual origin of intimal cells in cardiac-allograft arteriosclerosis. N Engl J Med.

[18] Gerthoffer WT (2007). Mechanisms of Vascular Smooth Muscle Cell Migration Circ Res.

[19] Han CI, Campbell GR, Campbell GH (2001). Circulating bone marrow cells can contribute to neointimal formation. J Vasc Res.

[20] Hillebrands JL, Klatter FA, van Dijk WD, Rozing J (2001). Origin of neointimal endothelium and alpha-actin-positive smooth muscle cells in transplant arteriosclerosis. J Clin Invest.

[21] Johnson P, Carpenter M, Hirsch GM, Lee TD (2001). Recipient cells form the intimal proliferative lesion in the rat aortic model of allograft arteriosclerosis. Am J Transpl.

[22] Saiura A, Sata M, Hirata Y, Nagai R, Makuuchi M (2001). Circulating smooth muscle progenitor cells contribute to atherosclerosis. Nat Med.

[23] Shimizu K, Sugiyama S, Aikawa M, Fukumoto Y, Rabkin E, Libby P (2001). Host bone-marrow cells are a source of donor intimal smooth-muscle like cells in murine aortic transplant arteriopathy. Nat Med.

[24] Grimm PC, Nickerson P, Jeffery J, Savani RC, Gough J, McKenna RM (2001). Neointimal and tubulointerstitial infiltration by recipient mesenchymal cells in chronic renalallograft rejection. N Engl J Med.

[25] Yeh ET, Zhang S, Wu HD, Korbling M, Willerson JT, Estrov Z (2003). Transdifferentiation of human peripheral blood CD34^+^-enriched cell population into cardiomyocytes, endothelial cells, and smooth muscle cells *in vivo*. Circulation.

[26] Bentzon JF, Weile C, Sondergaard CS, Hindkjaer J, Sassem M, Falk E (2006). Smooth muscle cells in atherosclerosis originate from the local vessel wall and not circulating progenitor cells in ApoE knockout mice. Arterioscler Thromb Vasc Biol.

[27] Wagers A, Sherwood RI, Christensen JL, Weissman IL (2002). Little evidence for developmental plasticity of adult hematopoietic stem cells. Science.

[28] Hu Y, Zhang Z, Torsney E, Afzal AR, Davison F, Metzler B (2004). Abundant progenitor cells in the adventitia contribute to atherosclerosis of vein grafts in ApoE-deficient mice. J Clin Invest.

[29] Ridley AJ, Schwartz MA, Burridge K, Firtel RA, Ginsberg MH, Borisy G (2003). Cell migration: Integrating signals from front to back. Science.

[30] Vicente-Manzanares M, Webb DJ, Horwitz AR (2005). Cell migration at a glance. J Cell Sci.

[31] Hall A (2012). Rho family GTPases. Biochem Soc Trans.

[32] Noma K, Oyama N, Liao JK (2006). Physiological role of ROCKs in the cardiovascular system. Am J Physiol Cell Physiol.

[33] Seasholtz TM, Majumdar M, Kaplan DD, Brown JH (1999). Rho and Rho kinase mediate thrombin-stimulated vascular smooth muscle cell DNA synthesis and migration. Circ Res.

[34] Negoro N, Hoshiga M, Seto M, Kohbayashi E, Ii M, Fukui R (1999). The kinase inhibitor fasudil (HA-1077) reduces intimal hyperplasia through inhibiting migration and enhancing cell loss of vascular smooth muscle cells. Biochem Biophys Res Commun.

[35] Liu B, Itoh H, Louie O, Kubota K, Kent KC (2002). The signaling protein Rho is necessary for vascular smooth muscle migration and survival but not for proliferation. Surgery.

[36] Sordella R, Jiang W, Chen GC, Curto M, Settleman J (2003). Modulation of Rho GTPase signaling regulates a switch between adipogenesis and myogenesis. Cell.

[37] Zhang L, Kloc M, Tejpal N, You J, Cordero-Reyes A, Youker KA (2012). Rock1 inhibitor abrogates chronic rejection in rat cardiac model system. Open J Organ Transplant Surg.

[38] Hidaka Y, Eda T, Yonemoto M, Kamei T (1992). Inhibition of cultured vascular smooth muscle cell migration by simvastatin (MK-733). Atherosclerosis.

[39] Rikitake Y, Liao JK (2005). Rho GTPases, statins, and nitric oxide. Circ Res.

[40] Lemstrom K, Sihvola R, Koskinen P (1997). Expression of platelet-derived growth factor in the development of cardiac allograft vasculopathy in the rat. Transplant Proc.

[41] Abid MR, Yano K, Guo S, Patel VI, Shrikhande G, Spokes KC (2005). Forkhead transcription factors inhibit vascular smooth muscle cell proliferation and neointimal hyperplasia. J Biol Chem.

[42] Beazley KE, Zhang T, Lima F, Pozharskaya T, Niger C, Tzitzikov E (2012). Implication for transglutaminase 2-mediated activation of β-catenin signaling in neointimal vascular smooth muscle cells in chronic cardiac allograft rejection. J Heart Lung Transplant.

[43] Wu D, Doods H, Stassen JM (2006). Inhibition of human pulmonary artery smooth muscle cell proliferation and migration by sabiporide, a new specific NHE-1 inhibitor. J Cardiovasc Pharmacol.

[44] Poon M, Marx SO, Gallo R, Badimon JJ, Taubman MB, Marks AR (1996). Rapamycin inhibits vascular smooth muscle cell migration. J Clin Invest.

[45] Sollott SJ, Cheng L, Pauly RR, Jenkins GM, Monticone RE, Kuzuya M (1995). Taxol inhibits neointimal smooth muscle cell accumulation after angioplasty in the rat. J Clin Invest.

[46] Goetze S, Xi XP, Kawano H, Gotlibowski T, Fleck E, Hsueh WA (1999). PPAR gamma-ligands inhibit migration mediated by multiple chemoattractants in vascular smooth muscle cells. J Cardiovasc Pharmacol.

[47] Tharaux PL, Bukoski RC, Rocha PN, Crowley SD, Ruiz P, Nataraj C (2003). Rho kinase promotes alloimmune response by regulating the proliferation and structure of T cells. J Immunol.

[48] Ohki S, Iizuka K, Ishikawa S, Kano M, Dobashi K, Yoshii A (2001). A highly selective inhibitor of Rho-associated coiled-coil forming protein kinase, Y-27632, prolongs cardiac allograft survival of the BALB/c-to-C3H/He mouse model. J Heart Lung Transplant.

[49] Mannon RB (2012). Macrophages: Contributors to allograft dysfunction, repair or Innocent bystanders?. Curr Opin Organ Transplant.

[50] Mosser DM, Edwards JP (2008). Exploring the full spectrum of macrophage activation. Nat Rev Immunol.

[51] Magil AB (2009). Monocytes/macrophages in renal allograft rejection. Transplant Rev (Orlando)..

[52] Wyburn KR, Jose MD, Wu H, Atkins RC, Chadban SJ (2005). The role of macrophages in allograft rejection. Transplantation.

[53] Hutchinson JA, Riquelme P, Sawitzki B, Tomiuk S, Miqueu P, Zuhayra M (2011). Immunological consequences and trafficking of human regulatory macrophages administered to renal transplant recipients. J Immunol.

[54] Fleming BD, Mosser DM (2011). Regulatory macrophages: Setting the threshold for therapy. Eur J Immunol.

[55] Ye Q, Wu YL, Foley LM, Hitchens TK, Eytan DF, Shirwan H (2008). Longitudinal tracking of recipient macrophages in a rat chronic cardiac allograft rejection model with noninvasive magnetic resonance imaging using micrometer-sized paramagnetic iron oxide particles. Circulation.

[56] Van Goethem E, Guiet R, Balor S, Charrière GM, Poincloux R, Labrousse A (2010). Macrophage podosomes go 3D. Eur J Cell Biol.

[57] Vérollet C, Charrière GM, Labrousse A, Cougoule C, Le Cabec V, Maridonneau-Parini I (2011). Extracellular proteolysis in macrophage migration: Losing grip for a breakthrough. Eur J Immunol.

[58] May RC, Machesky LM (2001). Phagocytosis and the actin cytoskeleton. J Cell Sci.

[59] Heasman SJ, Ridley AJ (2010). Multiple roles for RhoA during T cell transendothelial migration. Small GTPases..

[60] Linder S (2012). Macrophages on the move: how podosomes contribute to immune cell movement. DGZ Cell News..

